# A G-quadruplex-binding compound showing anti-tumour activity in an *in vivo* model for pancreatic cancer

**DOI:** 10.1038/srep11385

**Published:** 2015-06-16

**Authors:** Stephan A Ohnmacht, Chiara Marchetti, Mekala Gunaratnam, Rachael J Besser, Shozeb M Haider, Gloria Di Vita, Helen L Lowe, Maria Mellinas-Gomez, Seckou Diocou, Mathew Robson, Jiri Šponer, Barira Islam, R Barbara Pedley, John A Hartley, Stephen Neidle

**Affiliations:** 1 UCL School of Pharmacy, University College London, London WC1N 1AX, UK; 2 UCL Cancer Institute, University College London, London WC1E 6BT, UK; 3 Central European Institute of Technology (CEITEC), Campus Bohunice, Kamenice 5, 625 00 Brno, Czech Republic; 4 Institute of Biophysics, Academy of Sciences of the Czech Republic, Kralovopolska 135, 612 65, Brno, Czech Republic

## Abstract

We report here that a tetra-substituted naphthalene-diimide derivative (MM41) has significant *in vivo* anti-tumour activity against the MIA PaCa-2 pancreatic cancer xenograft model. IV administration with a twice-weekly 15 mg/kg dose produces ca 80% tumour growth decrease in a group of tumour-bearing animals. Two animals survived tumour-free after 279 days. High levels of MM41 are rapidly transported into cell nuclei and were found to accumulate in the tumour. MM41 is a quadruplex-interactive compound which binds strongly to the quadruplexes encoded in the promoter sequences of the BCL-2 and k-RAS genes, both of which are dis-regulated in many human pancreatic cancers. Levels of BCL-2 were reduced by ca 40% in tumours from MM41-treated animals relative to controls, consistent with BCL-2 being a target for MM41. Molecular modelling suggests that MM41 binds to a BCL-2 quadruplex in a manner resembling that previously observed in co-crystal structures with human telomeric quadruplexes. This supports the concept that MM41 (and by implication other quadruplex-targeting small molecules) can bind to quadruplex-forming promoter regions in a number of genes and down-regulate their transcription. We suggest that quadruplexes within those master genes that are up-regulated drivers for particular cancers, may be selective targets for compounds such as MM41.

Pancreatic cancer is among the most challenging to treat of all human cancers^1^. The majority (ca 95%) are classified as ductal adenocarcinomas, and of the others, the majority are neuroendocrine in origin. Tumours are associated most strongly with k-RAS mutations in almost all patients^2^. Mutated P53 and mutations in several other oncogenes such as CDKN2 are frequently observed^1^. Pancreatic cancer is not a rare cancer, with an estimated 45,000 new diagnoses in 2013 in the USA^3^ and ca 7,500 in the UK^4^; its incidence is increasing world-wide, with an estimate of ca 338,000 cases in 2012^4^. The prognosis, in striking contrast to other cancers, has barely changed in 40 years, with a 3–4% five-year survival rate^4^,^5^. The current standard drug treatment uses gemcitabine, a 2’-difluorinated pyrimidine analogue^6^. However it results in only very modest increases in median life expectancy due to the onset of chemo-resistance. A number of variants on the use of gemcitabine have been explored, including pro-drug approaches and combinations with paclitaxel^7^,^8^,^9^. However to date none have shown major improvements in late-stage clinical trials, which contrasts with the advances in treatment seen with a number of other cancer types.

We present here an alternative therapeutic strategy based on the targeting of genomic DNA, not at the duplex DNA level as with conventional cytotoxic agents, but at higher-order quadruplex loci within particular genes. Quadruplex nucleic acids are four-stranded arrangements that are formed from tandem repeats of short guanine-tracts and are structurally and functionally very distinct from normal duplex DNA (or RNA)^10^. Quadruplex-forming sequences are widely prevalent in the human (and other) genomes^11^,^12^, and are over-represented in promoter and untranslated (especially 5’-UTR) regions^13^,^14^. They are also over-represented in regions of genomic damage in cancer cells, for example at translocation hot-spots^15^,^16^. The existence of quadruplex DNA and RNA in human cells has been directly visualised using a quadruplex-specific antibody approach^17^,^18^, which has also shown that quadruplexes are significantly more prevalent in primary tumours compared to normal tissues^19^. In principle quadruplex structures, which are probably more transient in normal cells, can be the target of small molecules, which in the case of promoter sequences, would stabilise a quadruplex fold and hinder effective transcription of the targeted gene(s)^20^,^21^.

We have previously reported on the structure-based design and cellular evaluation of a series of tetra-substituted naphthalene diimide compounds as quadruplex-binding ligands^22^,^23^,^24^,^25^,^26^,^27^. One such compound (MM41: Fig. 1) has been characterised as a potent stabiliser of various quadruplex sequences and inhibitor of cell growth in a panel of cancer cell lines^28^. In particular it shows selectivity for several pancreatic cancer lines and its IC_50_ value (the concentration required to inhibit cell growth by 50% in a 96 hr assay), is 10 nM in the MIA PaCa-2 pancreatic adenocarcinoma cell line. This compound is not an inhibitor of telomerase activity *in vitro* or in cells and profiles of changes in gene expression from microarray experiments indicate that its mode of action is selective and does not involve conventional DNA damage responses or changes in telomere maintenance. Significant changes in the expression of the anti-apoptotic gene BCL-2 were noted from the cell-based experiments. BCL-2 was one of the most down-regulated of the genes examined in several gene array libraries^28^. This gene has well-characterised quadruplex-forming sequences in its promoter region^29^,^30^, as well as in its 5’-UTR^31^, which have previously been targeted by several distinct types of quadruplex-binding small molecules^32^,^33^. it is also apparent that some small molecules can target the complementary i-motif strand in the BCL-2 promoter^34^.

We report here on the anti-tumour activity of the naphthalenediimide compound MM41 (Fig. 1), in a MIA PaCa-2 xenograft model for human pancreatic cancer. Effects on BCL-2 and k-RAS protein expression in treated tumours are reported, together with data on cellular localisation. MM41 binds especially strongly *in vitro* to the BCL-2 quadruplex and the features of a molecular model of the complex have also been examined. These are interpreted in terms of the generic features of the compound’s ability to bind to quadruplex DNAs in general.

## Results

### MM41 interacts with the BCL-2 and other promoter quadruplexes

Fluorescence resonance energy transfer (FRET) melting methods have been used to characterise the ability of MM41 to stabilise several distinct intramolecular DNA quadruplexes. It has been previously reported that this compound stabilises a human telomeric quadruplex sequence. Studies with two distinct quadruplex sequences from the k-RAS promoter and one from the BCL-2 promoter (Table 1) show that MM41 has some selectivity for the BCL-2 promoter quadruplex over the two k-RAS ones. However MM41 also binds effectively to other quadruplexes^28^.

**Figure 2:**
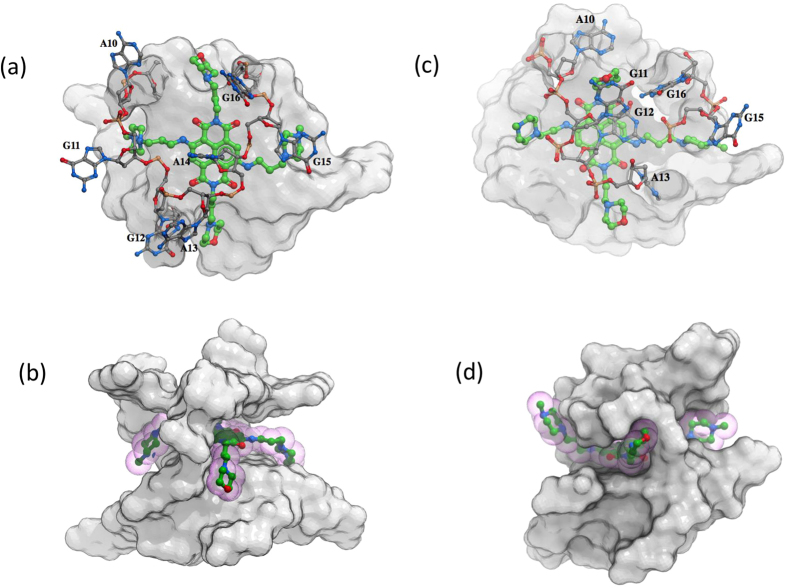
Visualisations of the binding of a MM41 molecule to a BCL-2 promoter quadruplex, taken from the molecular modelling study. (**a**) Top view of the quadruplex with the MM41 molecule involved in π-π interactions with the terminal G-quartet. (**b**) Side view of the docked complex. The four side-chain arms of MM41 penetrate into the four grooves of the quadruplex; however, the side-chains are not long enough to be involved in direct interactions with the backbone. (**c**) Top view of the final snapshot from the MD simulations. Base G12 forms π-stacking interactions with the chromophore of MM41, which is then sandwiched between G12 and the top G-quartet. (**d**) Side view of the final snapshot from the MD simulations illustrating the rearrangement of loop-2 nucleotides, which now interact with the side chains of MM41. The drug is shown as green sticks while loop-2 surface is shown in cpk colours.

Previous crystallographic studies have revealed the structural details of MM41 binding to a human telomeric quadruplex^27^,^28^. No experimental data on the structure of MM41 complexes with other quadruplexes is currently available, so molecular dynamics simulation methods have been used to examine the likely binding features of a modelled BCL-2 quadruplex complex (Fig. 2a,b). The loops in the native BCL-2 parallel quadruplex derived from the NMR model^29^ are highly mobile, particularly the ones where MM41 binds. A long time-scale molecular dynamics simulation (>100 ns) using the ParmBSc0 force-field^35^ resulted in a configuration with a MM41 molecule being stacked on the loop bases, which is unlikely to be in accord with the high experimental ΔT_m_ value and is not in accord with the crystallographic observations of ligand chromophore stacking onto a terminal G-quartet of a quadruplex^27^,^28^. The parmXOL4 (XOL4) modification^36^ of the Cornell *et al.* force-field was then used, which refines the *syn*-*anti* glycosidic angle balance and facilitates the *syn*-*anti* transition through the 120° X region by decreasing the energy barrier for this transition and increasing it through the 350° X region. This resulted in the loops being much more structured than before and critically the bound MM41 molecule is now in a stereo-chemically more reasonable binding position, with the core chromophore in π-π stacking contact with the terminal G-quartet of the quadruplex and the four arms of the compound in close non-bonded contact with the loops. In this model residue G12 is swung round and stacks over the chromophore, which is thus sandwiched between the terminal quartet and G12.

### MM41 shows anticancer activity *in vivo*

The maximum tolerated dosage (MTD) for iv administration of MM41 was found to be ca 30 mg/kg. A preliminary pharmacokinetic study at 20 mg/kg has determined the *in vivo* half-life to be ca 4 hrs (Supplementary Table S1 and Figure S1). Since the targets (individual genomic DNA quadruplexes) are present in low copy numbers per cell, this half-life value suggests that even with twice-weekly dosing of MM41, there would be sufficient bioavailability to produce transcriptional inhibition of particular genes by means of quadruplex stabilisation. A therapeutic schedule of two doses were explored with the MIA PaCa-2 pancreatic tumour xenograft model, conducted at 10 and 15 mg/kg, each twice weekly, over a period of 5½ weeks (40 days: 12 doses). At this point the mice were sacrificed, apart from two from the 15 mg/kg group, which were maintained for a further 239 days without any further MM41 dosing. These two mice were chosen since their xenografts had completely regressed during the dosage period.

**Figure 3:**
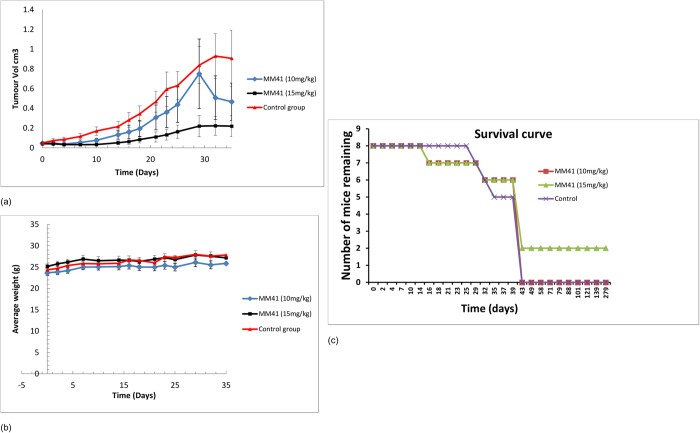
(**a**) Efficacy of MM41 in subcutaneous tumor xenografts, evaluated in terms of tumour growth over time ± SEM. Control mice are compared to groups dosed with 10 and 15 mg/kg respectively, administered twice daily. The change in tumour volume for the group dosed with 15 mg/kg of MM41 is statistically significant (Student’s t-test) relative to the control group with P = 0.026 (n = 6). (**b**) Average mouse weight over time for the three experimental groups of mice. (**c**) Kaplan-Meier survival plot for the three groups of mice. Note that cessation of particular experiments at day 40 (see text) accounts for the zero survival of 2/3 groups at this time-point.

MM41 shows a dose-dependent anti-tumour response, with the higher 15 mg/kg dose producing a significantly greater effect than the 10 mg/kg regimen. At 40 days, the conclusion of the main therapeutic dosage experiment, an average of ca 80% decrease in tumour growth was observed over the group of six animals used (Fig. 3a). From 30 days onwards, tumour regrowth ceased. No significant weight reduction or adverse effects such as tumour ulceration were observed in either group at any time during the course of the experiments (Fig. 3b). Within the 15 mg/kg group the tumours in two mice were observed to have completely regressed and no sign of tumour regrowth was observed during the subsequent 239 days (Fig. 3c), at the end of which the mice showed some signs of normal aging and were sacrificed. Histopathological examination of liver, spleen and kidney tissues from control and treated animals showed no signs of tissue damage and are consistent with normal histology for these organs (data obtained by Mohammed Rashid, UCL Cancer Institute).

### MM41 localises in cell nuclei and in tumours

**Figure 4:**
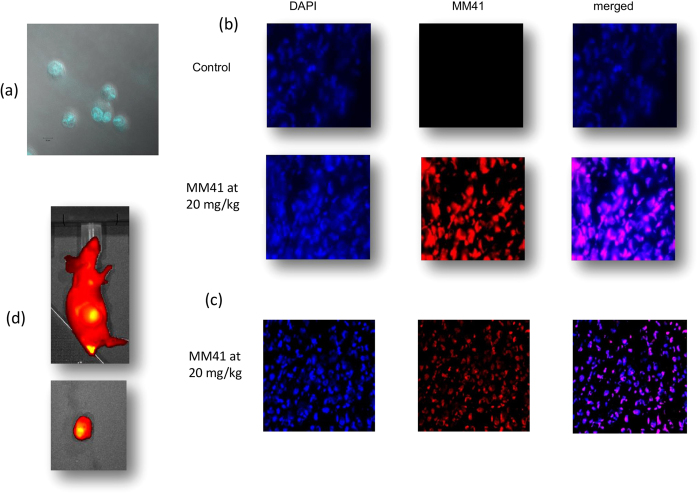
(**a**) Confocal image of MIA PaCa-2 cells following a 30 min exposure to MM41. The fluorescence of the compound is seen concentrated in the cell nuclei. (**b**) Images (x 40) of tumour tissue from control and MM41-treated MIA PaCa-2 xenografts, showing DAPI-stained and MM41 fluorescent images, together with merged images showing superposition of DAPI and MM41 staining. The images are of sections close to the tumour surface. (**c**) Images (x 20) showing DAPI and MM41 staining, together with the merged image, for sections taken from the centre of a treated tumour. (**d**) (LHS) IVIS whole-body fluorescence image, taken at the MM41 emission wavelength, showing that MM41 is concentrated in the tumour, which is on the flank of the animal. A substantial amount of MM41 is shown to be retained at the site of administration. An image of the extracted tumour is also shown.

The high intrinsic fluorescence of MM41, with emission at ca 660 nm, has readily enabled cellular and tumour uptake to be monitored. Figure 4a shows that MM41 is rapidly taken up into nuclei of cultured MIA PaCa-2 cells and after 30 min, almost all of the fluorescence is in the cell nuclei. Figure 4b shows images from tumour tissues taken from control and treated animals, 4 hrs after MM41 administration. These show (i) that MM41 is exclusively localised in cell nuclei, co-localising with DAPI (Fig. 4b) and (ii) that MM41 penetrates into the centre of the tumour mass (Fig. 4(c)). Whole-animal imaging using IVIS (PerkinElmer) technology (Fig. 4d) showed that MM41 is preferentially localised in the tumour mass 4 hr post-administration, and only background fluorescence was apparent in other body regions.

### BCL-2 and k-RAS are down-regulated

**Figure 5:**
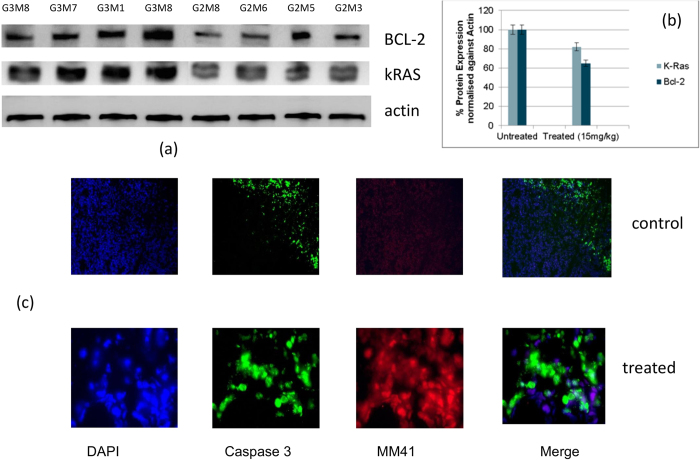
(**a**) Western blots for BCL-2 and k-RAS proteins, and actin as a control. The four LHS columns represent blots from four untreated mice, and the four RHS ones are from four MM41-treated (at 15 mg/kg). All mice were taken at the termination of the therapeutic experiments. (**b**) Plot of the averaged changes in BCL-2 and k-RAS proteins, normalised against the actin blots. (**c**) Images (X 10) of caspase immuno-staining in sections from control and MM41-treated tumours.

Western blots of BCL-2 protein and k-RAS enzyme for four animals show a consistent pattern of reduction in protein levels for all four treated tumours that were examined, compared to controls (Fig. 5a). This comparison can only be qualitative since comparison with an actin standard shows that levels in the control tumours (and by implication in the treated ones as well) are variable; the averaged change is 40% (Fig. 5(b)). Changes in k-RAS are less, and average 30%. Evidence for apoptosis was sought, using a caspase 3 antibody. Figure 5(c) shows that treated tumours, but not untreated ones, show high levels of caspase 3 staining, consistent with induction of apoptosis.

Changes in expression levels for several other proteins such as c-MYC, for which promoter quadruplex sequences have been identified and shown to form stable quadruplexes, at least *in vitro*^21^, were also examined, but no significant changes were observed (data not shown). Possible changes in the activity of telomerase in treated tumours were also investigated using a modified TRAP assay^37^; no significant changes in the enzymatic activity of telomerase were found (data not shown).

## Discussion

We show here that the tetra-substituted naphthalene diimide quadruplex-binding compound MM41 has significant anti-tumour activity in the MIA PaCa-2 pancreatic cancer xenograft model, and that two treated animals showed curative responses, with no tumour regrowth seen even after >200 days post-treatment. The design strategy for developing the MM41 molecule was to down-modulate cationic charge by substituting two of the four N-methyl-piperazine groups in the 1^st^-generation compound by morpholino ones^28^. The approach was supported by comparing the present results from those in a previous pancreatic cancer xenograft study with the earlier-generation ND compound having four substituent N-methyl-piperazine groups^26^. This compound showed significantly lower anti-tumour activity, in accordance with its 10-fold lower cell growth inhibitory activity in pancreatic cancer cells compared to MM41.

MM41 has previously been shown to have exceptional anti-proliferative activity in several pancreatic cell lines, with an IC_50_ value of 10 nM in the MIA PaCa-2 line^28^. MM41 is also a potent binder *in vitro* to a range of quadruplex structures, including those encoded by sequences in the human BCL-2 and k-RAS promoters. The present study finds that levels of the BCL-2 and k-RAS proteins are both reduced in treated tumours relative to untreated control tumours and apoptosis is induced, providing correlative evidence that these genes are targets of MM41 *in vivo*. The fact that levels of the anti-apoptotic protein BCL-2 are only reduced on average by 40% suggests that either there are other as yet undiscovered targets of MM41, or possibly that this level of BCL-2 decrease is sufficient to drive tumour cells to apoptosis. It is known that MM41, in common with the majority of other quadruplex-binding compounds, does not appear to have specificity for a single target quadruplex–MM41 binds tightly to most other quadruplexes that we have examined. This is unsurprising since the modelling study reported here shows that the MM41-BCL-2 quadruplex complex has many features in common with the bound MM41 in the crystal structure of the MM41-human telomeric quadruplex.

It is not clear at present why then MM41 does not affect telomere-maintenance pathways, in striking contrast with the behaviour of a number of quadruplex-binding compounds whose mechanism of action involves stabilisation of telomeric quadruplexes at the 3’ end of telomeres, uncapping of telomeric proteins and the induction of a DNA-damage response^38^,^39^,^40^,^41^. We suggest that quadruplexes within the driver genes (such as k-RAS) in Mia-Paca2 tumours, may be especially susceptible to MM41 action. BCL-2 expression is up-regulated in the majority of primary pancreatic tumours^42^ and has been targeted at the protein level by a range of small molecules, including the BH3 domain mimeric GX15-070 (obatoclax)^43^ and the pan-BCL-2 inhibitor (-)-gossypol^44^, also known as AT-101, although such agents have most often been investigated in combination with other therapies. A siRNA approach for silencing BCL-2 expression has demonstrated BCL-2 down-regulation and tumour regression in the PANC-1 pancreatic cancer xenograft model^45^. The results presented here suggest that apoptosis induction via BCL-2 inhibition, probably at the transcriptional level, may be a significant contributor to the anticancer activity of MM41. It is not possible to rule out other quadruplex promoter (or mRNA) targets and it is plausible that the effectiveness of MM41 is because it is able to affect a number of key driver genes. The BCL-2 promoter region is guanine-rich and several potential quadruplex-forming sequences within this promoter have been identified^29^,^30^,^46^ including the one studied here. In addition, a quadruplex-forming sequence in the 5’-UTR of BCL-2 has been reported and we are therefore unable to rule out the possibility that MM41 is regulating BCL-2 expression at the translational level. However its rapid localisation in the nuclei of MIA PaCa-2 cells both in culture and in the tumour mass, support the concept that the quadruplex target(s) are nuclear rather than cytoplasmic.

MM41 itself is not an ideal drug molecule in the standard medicinal chemistry sense. It has a high molecular weight, carries four positive changes (although the two morpholino groups are not strongly basic) and does not for example, obey Lipinski’s rules. However it is readily formulated as a water-soluble salt, ensuring that it is sufficiently bioavailable. It also does not have significant hERG or CYP450 liabilities (Table S2) and does not damage vital organs in mice, altogether suggesting that this class of naphthalene diimide derivatives is worthy of further study for possible therapeutic use in humans. There is no current data on the mechanism of cellular uptake for MM41 and similar large polycationic quadruplex-binding ligands, but it is known that existing anti-neoplastic drugs such as mitoxantrone are effectively transported into tumour cells by the human plasma membrane protein, the organic cation transporter OCT1. The preliminary confocal microscopy studies reported here demonstrate that the large size and relatively high molecular weight of MM41 is not a hindrance to rapid nuclear uptake, possibly via OCT1. It is also encouraging to note that examination of the interior of the xenograft tumour mass also shows significant MM41 uptake, which may well be a major factor in the complete tumour regression which was observed. It is notable that MM41 fluorescence appears to completely fill each cell nucleus from treated tumours (Figs 4(a–c)), whereas in principle if MM41 is targeting a small number of nuclear quadruplexes one might expect to see just individual foci, analogous to what has been observed with quadruplex-specific antibodies^17^. The fluorescence is likely to be a consequence of the concentration of MM41 being relatively high so any individual MM41-bound quadruplex foci signals would be swamped by the high fluorescence background of MM41. In any case the resolution of these images would be highly unlikely to adequately resolve individual MM41-quadruplex foci.

The modelling studies show that its plausible binding mode to the BCL-2 quadruplex is closely analogous to that observed in the crystal structures of the MM41 complex with a human telomeric quadruplex and recent modelling predictions^47^, providing support for the concept that MM41 is able to bind effectively to a number of quadruplex targets, and thus making it an effective poly-quadruplex agent. A trisubstituted ND derivative has been recently found^48^ to affect the expression of several genes including BCL-2, although by contrast with the present study, changes in protein levels were not observed. The poly-quadruplex targeting ability of MM41 may well be significant for its effectiveness *in vivo* as an anti-tumour agent.

## Methods

The synthesis and characterisation of MM41 (4,9-bis((3-(4-methylpiperazin-1-yl)propyl)amino)-2,7-bis(3-morpholinopropyl) benzo[*lmn*][3,8] phenanthroline-1,3,6,8(2H,7H)-tetraone: molecular weight 831.08) has been previously reported^23^,^28^. MM41 was analytically pure as shown by lc-ms and NMR methods and was formulated for biological studies as the freely water-soluble formate salt.

### FRET studies

FRET DNA melting assays on MM41 were performed using a fluorescence resonance energy transfer (FRET) assay modified as a high-throughput screen in a 96-well format.^49^ The labelled oligonucleotides had attached the donor fluorophore FAM: 6-carboxyfluorescein and the acceptor fluorophore TAMRA: 6-carboxytetramethyl-rhodamine. The FRET probe sequences were diluted from stock to the correct concentration (400 nM) in a 60 mM potassium cacodylate buffer (pH 7.4) and then annealed by heating to 95 °C for 10 min, followed by cooling to RT in the heating block (3–3.5 hrs). Solutions (at 2x concentration) were prepared using 60 mM potassium cacodylate buffer (pH 7.4). 96-well plates (MJ Research, Waltham, MA) were prepared by aliquoting 50 μl of the annealed DNA into each well, followed by 50 μl of the compound solutions. Measurements were made on a DNA Engine Opticon (MJ Research) with excitation at 450–495 nm and detection at 515–545 nm. Fluorescence readings were taken at intervals of 0.5 °C in the range 30–100 °C, with a constant temperature being maintained for 30 sec prior to each reading to ensure a stable value. Final analysis of the data was carried out using a script written in the program Origin 7.0 (OriginLab Corp., Northampton, MA). The advanced curve-fitting function in Origin 7.0 was used for calculation of ΔT_m_ values. Esds in ΔT_m_ are ± 0. °C.

### Molecular dynamics simulations

The starting point for the modelling study was the NMR structure of the BCL-2 promoter quadruplex, with mixed parallel/antiparallel G-strands forming the core of the quadruplex^29^, and a docked MM41 molecule (details of the docking procedure are given in the Supplementary Information). The G-stem bases exhibit both *syn* and *anti*-orientations. Previous studies have shown that parmX_OL4_ (X_OL4_) modification of the Cornell *et al.* force field refines the *syn*-*anti* balance as it facilitates *syn-anti* transitions through the 120° X region by decreasing the energy barrier for this transition, increases it through the 350° X region and refines the shape and depth of the *syn* minimum^36^. Previous simulations of G-quadruplexes in X_OL4_ have also shown an improvement in structures with respect to the simulations with earlier force field versions^36^. The X_OL4_ refinement has recently been included as DNA default force field in the AMBER code. Therefore, we carried out the present simulations using the parmbsc0 version of the Cornell et al. force-field with the (X_OL4_) modifications^35^,^36^,^50^,^51^ Solvation and addition of more ions to the quadruplex were performed with the help of the xleap module of the AMBER12 program (Case, D. et al. AMBER 12. University of California, San Francisco, 2012). The quadruplex was neutralized using K^+^ ions and TIP3P water molecules were used for solvation. The system was placed in a periodic box whose boundaries extended at least 10 Å from any solute atom. The parameters for K^+^ ions were adapted for AMBER from a previous study (radius 1.593 Å and well depth 0.4297054 kcal mol1)^52^. The parameters for the MM41 ligand were generated via the Antechamber module of the AMBER software using the GAFF force field.

Standard equilibration protocols were used for initial minimization of the structure. The first round of equilibration with explicit solvent and ions involved 1000 steps of steepest descent, followed by 1000 steps of conjugate gradient energy minimization. A 300 ps MD equilibration was performed in which the quadruplex was constrained, whereas the solvent and ions were allowed to equilibrate. The system was gently heated from 0 K to 300 K with a time step of 0.5 ps. This was followed by subsequent rounds of MD simulation, at constant pressure and 300 K for 1 ns. The constraints were gradually relaxed, until no constraints were applied to the system. The final MD simulations were carried out for 350 ns using ACEMD^53^. The periodic boundary conditions were defined by the PME algorithm and non-bonded the cut-off was set to 10 Å^54^. Covalent bonds involving hydrogen atoms were constrained using the SHAKE algorithm with a tolerance of 0.0001 Å, which allowed the use of an integration time step of 2 fs^55^. All the simulations were carried out at constant pressure of 1 atm and constant temperature of 300 K. The temperature and pressure was maintained using a Berendsen weak coupling thermostat^56^. The final production run without restraints was carried out for a continuous 350 ns and the frames were collected every 20 ps. Analyses of trajectory were performed using the ptraj module of AMBER^57^ and the VMD^58^ and PYMOL (DeLano, W. L. The PyMOL Molecular Graphics System, DeLano Scientific, San Carlos, CA, USA, 2002) programs were used for visualization.

### Confocal studies

The human cancer cell line, MIA PaCa-2 (derived from a human pancreatic carcinoma), was purchased from ATCC. Cells were maintained in DMEM culture medium, supplemented with 10% fetal bovine serum, 2 mM L-glutamine and 1:100 dilution of penicillin-streptomycin solution Hybri-MaxTM. The cell line was maintained at 37 °C, 5% CO_2_ and routinely passaged. Cells were seeded on poly-D-lysine coated coverslips in 12-well plates, 24 hours prior to addition of anticancer compound. To prepare the coverslips they were sterilised with 100% ethanol and rinsed with 1X PBS. Coverslips were then coated with 25 μg/ml poly-D-lysine for 1 hr at room temperature, rinsed in 1X PBS, and left to fully dry in the Laminar Flow Cabinet overnight before use. MM41 was added to the cells at a concentration of 333.2 nM (equivalent to 20 X IC_50_) in culture medium. Following a 30 min incubation period with MM41 at 37 °C, cells were rinsed with 1 x PBS and then fixed with ice-cold methanol for 15 min at RT. The fixed cells were washed twice with ice cold 1 x PBS. Coverslips were then mounted with a drop of vectashield mounting medium to microscope slides. Coverslips were sealed in place with nail polish. Trans light confocal images were obtained with a Zeiss LSM 710 microscope. Fluorescence from MM41 was collected with a 633 nm HeNe laser. Cells were imaged using a 63 x oil immersion lens.

### Pharmacokinetics studies

A single dose of 20 mg/Kg was administered intravenously to mice and 25 μl blood was drawn from the tail vein at time-points 10, 20, 30, 60, 120, 240, 360 and 1440 mins. These samples were mixed immediately with 225 μl phosphate buffered saline and centrifuged for 5 min at 21,000 × g. 200 μl supernatant was transferred to a cryovial and frozen at –70 °C until analysis.

Plasma samples were thawed and the MM41 was extracted using SOLA HRP 10 mg/ml cartridges (Thermo Scientific) according to manufacturer’s instructions. Samples were dried under nitrogen and reconstituted in 200 μl mobile phase (0.1% trifluoroacetic acid in H_2_O and 0.1% trifluoroacetic acid in acetonitrile). High performance liquid chromatography was performed using a C18 reversed phase column with a water (0.1% TFA)/ acetonitrile (0.1% TFA) gradient (0–2 min 10% acetonitrile, 2–3 min 10%–30% acetonitrile, 3–6 min 30%–80% acetonitrile, 6–9 min 80% acetonitrile, 9–10 min 80%–10% acetonitrile, 10–12 min 10% acetonitrile). The flow rate was 1 ml/min and the column oven was set to 40 °C. MM41 was detected by its fluorescence, with an excitation wavelength 280 nm and an emission wavelength 660 nm. The standard curve range was 100–10,000 nM.

### Xenograft studies

All animal experiments were performed in accordance with the UK Home Office Animals Scientific Procedures Act 1986, and United Kingdom Co-ordinating Committee on Cancer Research Guidelines for the Welfare and Use of Animals in Cancer Research^59^ and with approval of the University College London Animal Ethics Committee.

The maximum tolerated dose (MTD) study of MM41 was performed in CD-1 nude mice. The MTD of a single dose was determined and repeat dosing was performed per week at different concentrations of MM41 (20, 25 and 30 mg/kg), administered intravenously (iv).

For therapy studies, female CD-1 nude mice (2–3 months old, weighing 20–25 g) were injected subcutaneously with 5 × 10^6^ Mia PaCa-2 cells in the right flank (unsupplemented RPMI + Matrigel). When the tumours were established (approx. 13 days, mean size 0.05 cm^3^), the mice were divided into three therapeutic groups with eight mice/group. The MM41 samples were dissolved directly in saline to the required concentrations and administered iv. Tumour size was measured weekly using the π-based ellipsoid volume formula^60^ (length × width × height × π/6) every 3–4 days, and the mice were also weighed and examined at the same time to determine any signs of toxicity from the drug.

Group 1: 8 mice treated with a twice weekly dose of 10 mg/Kg of MM41 in saline.

Group 2: 8 mice treated with a twice weekly dose of 15 mg/Kg of MM41 in saline.

Group 3: 8 control mice treated with saline only, twice weekly.

Mice were culled if tumours ulcerated, if tumours reached a size of 1.5 cm^3^, or if a weight loss of 10–20% of the initial body weight was observed. The plot in Fig. 3a for 10 mg/kg dosing drops down at around day 28 as mice were culled due to them exceeding the tumour size limits on the project licence and this resulted in a temporary group average lowering, as seen on the Figure. Similarly there is an apparent decrease in average tumour volume at day 32 for the control group of mice, which is not a real effect but is also a consequence of the tumours in some animals reaching the maximum permitted size.

### Imaging studies

Mice were anesthetised using Isoflurane and imaged using the IVIS pre-clinical *i*maging system from Perkin Elmer. Images were captured at the following time points 30 min, 1 hr, 2 hr, 4 hr, 8 hr after intravenous administration of MM41using a Cy5.5 excitation for 5 sec.

### Western blot analysis

Tumour samples from four treated and four control animals at the termination of the therapeutic xenograft experiments were excised into small pieces and frozen in liquid nitrogen. Approximately 5 mg of each tumour was lysed in RIPA lysis buffer (1x) containing protease cocktail inhibitors, PMSF and sodium orthovanadate (Santa Cruz Biotechnology) according to the manufacturer’s instruction. Total protein concentrations were determined using the Pierce BCA protein assay kit according to the manufacturer’s instructions. Total protein from the samples were loaded onto pre-cast SDS-PAGE gels (Bio Rad) and transferred onto a nitrocellulose membrane (Invitrogen) and the membranes were probed with primary antibodies against BCL-2, k-RAS and β-actin (Santa Cruz Biotechnology). Following incubation with the appropriate secondary antibodies the membranes were visualized with the horseradish peroxidase luminescent visualisation system (National Diagnostics).

### Apoptosis immuno-staining

Mice received the 12^th^ dose of MM41 on day 39 and were culled on day 41 of the treatment starting-point. Tumours from mice M5 and M6 of group 2 (treated with 15 mg/kg of MM41) and mouse M1 from group 3 (control) were snap-frozen and cut in 10 μm sections, then stained with either purified rabbit anti-activated caspase 3 or purified rabbit anti-BCL-2 overnight at 4 °C then with a goat anti-rabbit coated with alexa-488 at room temperature for 2 hr. Slides were counterstained with DAPI nuclear marker (1 μg/mL) for 30 min at RT. Images were taken with a Zeiss fluorescence microscope and analysed with Image J software.

## Additional Information

**How to cite this article**: Ohnmacht, S. A. *et al.* A G-quadruplex-binding compound showing anti-tumour activity in an *in vivo* model for pancreatic cancer. *Sci. Rep.* **5**, 11385; doi: 10.1038/srep11385 (2015).

## Supplementary Material

Supplementary Information

## Figures and Tables

**Figure 1:**
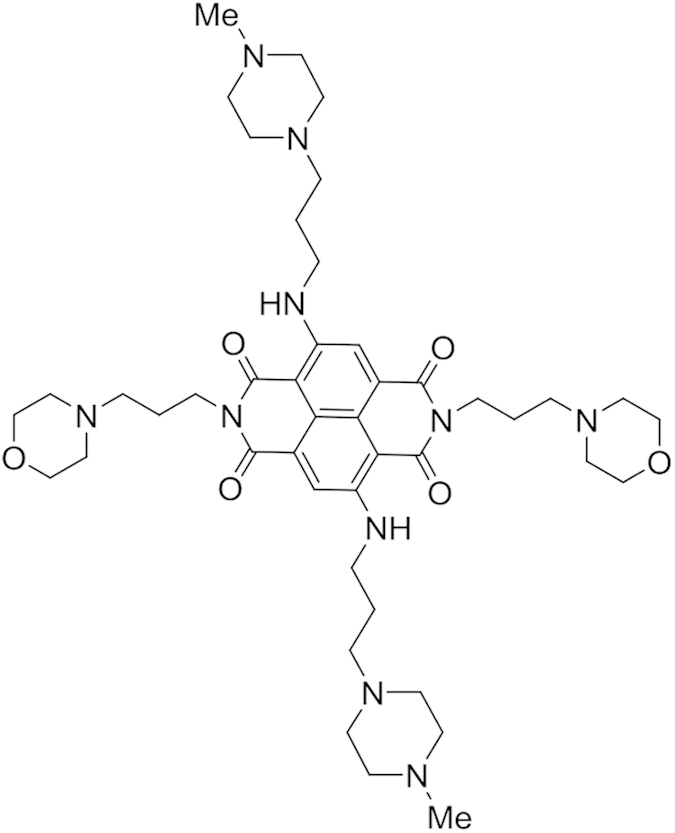
The structure of MM41.

**Table 1:** Increase in melting temperature (ΔTm values in °C) from FRET experiments with MM41 at 1 μM concentration, and three quadruplex promoter sequences, from the k-RAS and BCL-2 genes. Esds are from triplicate measurements and average ±0.1 °C.

**k-RAS1**	**k-RAS2**	**BCL-2**
22.5	19.8	26.4
