# Supplementary material for: A G-quadruplex-binding compound showing anti-tumour activity in an in vivo model for pancreatic cancer
Source: Sci Rep. 2015 Jun 16;5:11385. doi: 10.1038/srep11385 (PMC4468576; doi:10.1038/srep11385)
Supplement: Supplementary Information [file srep11385-s1.pdf]

Supplementary Data

A G-quadruplex-binding compound showing anti-tumour activity in an in vivo model for pancreatic cancer

Stephan A Ohnmacht, Chiara Marchetti, Mekala Gunaratnam, Rachael J Besser, Shozeb M Haider, Gloria Di Vita, Helen L Lowe, Maria Mellinas-Gomez, Seckou Diocou, Mathew Robson, Jiri Šponer, Barira Islam, R Barbara Pedley, John A Hartley and Stephen Neidle*

Time (min)	Mouse 1 Concentration (nM)
0	0
10	1655
20	1613.5
30	1512.23
60	1460
120	1033
240	861.6
360	792
1440	Bld*

* - below limit of detection

Table S1 Concentration of MM41 in each sample as determined by fluorescence HPLC, during the pharmacokinetic study.

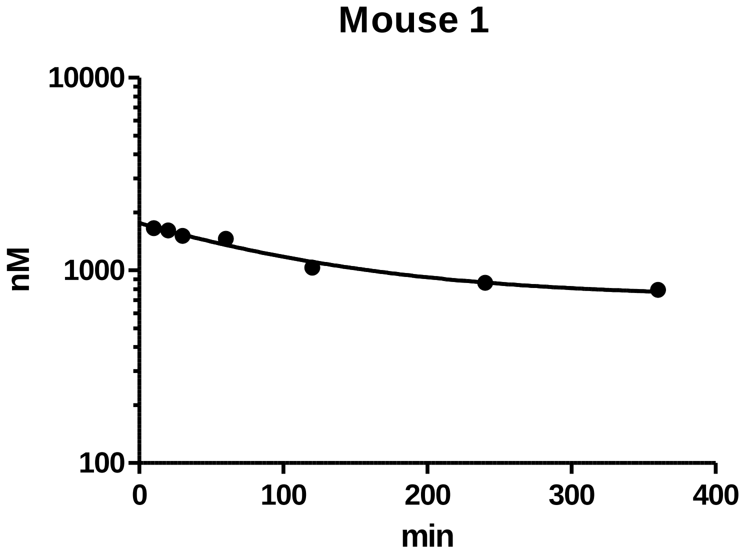

Figure S1. Plot of the pharmacokinetic behaviour of MM41, for one of the animals used in the study.

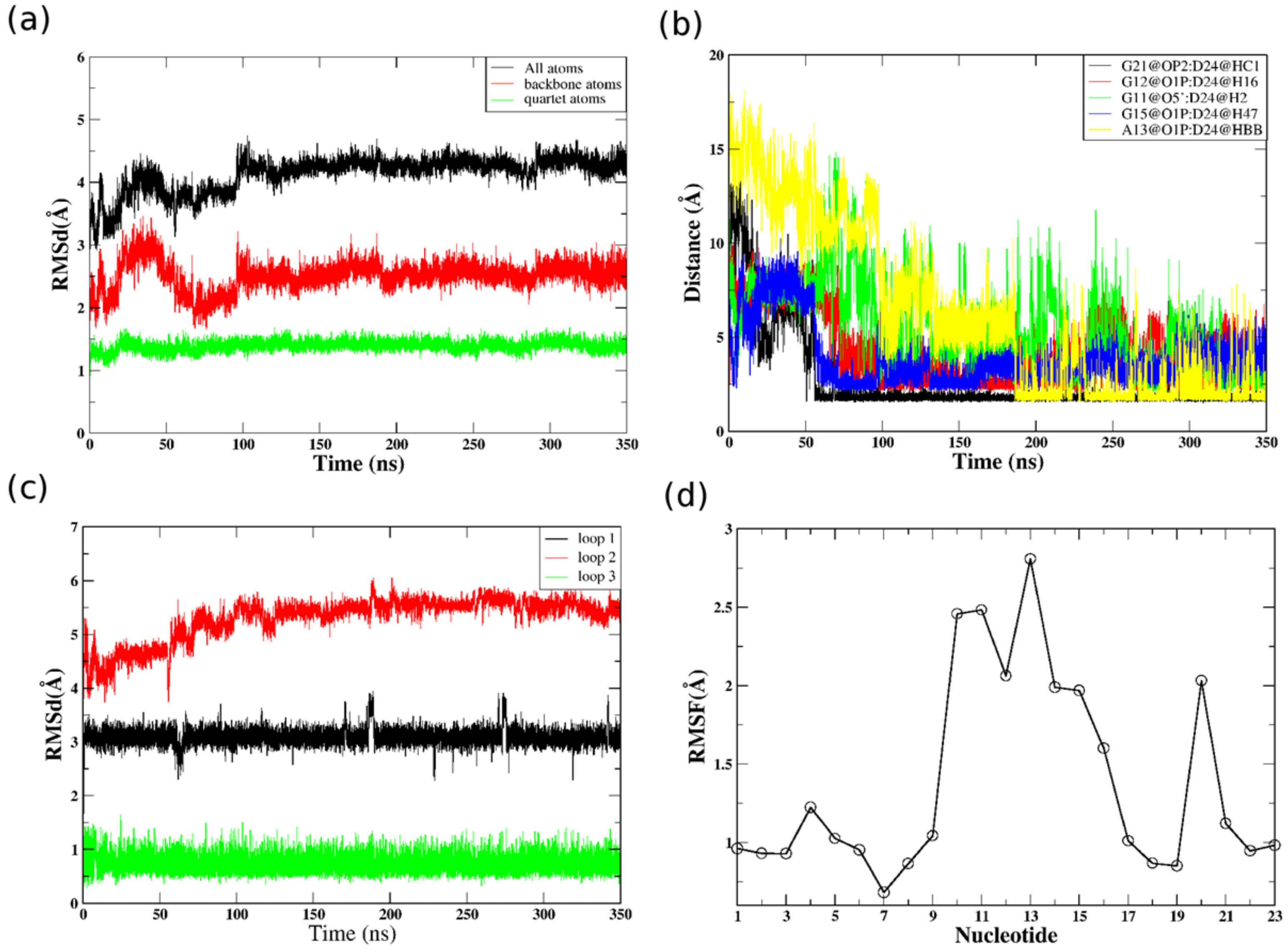

Figure S2 (a) Root mean square deviation (RMSd) analysis of the BCL-2 promoter quadruplex molecular dynamics simulation showing all atoms, backbone and quartet atom RMSds in the 350 ns simulation (b) Interaction of MM41 (D24) atoms with the backbone atoms of the BCL-2 quadruplex as observed in the 350 ns long simulation. The RMSd of the backbone is less flexible as the drug forms stable interactions with the backbone atoms (c) RMSd comparison of the loops highlights that loop-2 is the most flexible loop of the quadruplex and (d) the average root mean square fluctuation of the BCL-2 promoter quadruplex simulation shows that loop residues (4-6, 10-16, 20) fluctuate more than the quartet residues. Amongst the loops, the long lateral loop-2 (residues 10-16) exhibits the highest RMSF values.

Methodology for Molecular Modelling Docking of MM41

The NMR structure of the Bcl-2 promoter quadruplex (PDB id 2F8U) was used as a starting structure for modeling studies. Two thymine nucleotides (Thy14, Thy15), in the loop, were modified to guanines to be consistent with the native Bcl-2 sequence of 5'-GGGCGCGGAGGAAGGGGGCGGG-3'. Mutations were carried out using the program X3DNA,[1,2] which replaces the nucleobases without altering the backbone conformation. The ligand-binding site was generated based on the protocol from Read et al.[3] The site was introduced between Loop-2 (Ade10-Gua16) and the top quartet by breaking two phosphodiester bonds (between Gua9-Ade10 and Gua16-Gua17) and separating the two halves of the structure such that the separation between the quartet and Ade12 was 6.8Å. The backbones were reconnected followed by 1000 steps of conjugate gradient energy minimization to relieve any structural distortions.

The chemical structure of MM41 ligand was built and docked using the ICM software package.[4] The charges were assigned to the ligand using the ICFF force field.[4] Grid maps were made around the binding site that encompassed all residues from the loop and the four guanines from the top quartet. Docking was carried out using the automated docking module of the ICM software. The docked conformation was chosen based on the highest binding energy. The docked complex was then used as a starting structure for molecular dynamics simulations. The four side chains of the MM41 ligand access the four grooves in the quadruplex structure.

1. Lu, X. J. & Olson, W. K. 3DNA: a versatile, integrated software system for the analysis, rebuilding and visualization of three-dimensional nucleic-acid structures. *Nature Prot*. **3**, 1213-1227 (2008).

2. Colasanti, A. V., Lu, X. J. & Olson, W. K. Analyzing and building nucleic acid structures with 3DNA. *J. Vis. Exp*. e4401 (2013).

3. Read, M., Harrison, R. J., Romagnoli, B., Tanious, F. A., Gowan, S. H., Reszka, A. P., Wilson, W. D., Kelland, L. R. & Neidle, S. Structure-based design of selective and potent G quadruplex-mediated telomerase inhibitors. *Proc. Natl. Acad. Sci. USA* **98**, 4844-4849 (2001).

4. Katritch, V., Totrov, M. & Abagyan, R. ICFF: a new method to incorporate implicit flexibility into an internal coordinate force field. *J. Comp. Chem*. **24**, 254-265 (2003).

Table S2. Selected receptor-binding data from assays run on MM41 by Eurofins (www.eurofins.co.uk), using standardised *in vitro* assay conditions.

Receptor	Species	Concentration	% inhibition
CYP450, 1A2	human	10 µM	8
CYP450, 2C19	human	10 µM	-8
CYP450, 2C9	human	10 µM	-5
CYP450, 2D6	human	10 µM	18
CYP450, 3A4	human	10 µM	-22
GABA, , Flunitrazepam, Central	Rat	10 µM	0
GABAA, Muscimol, Central	Rat	10 µM	10
Glutamate, NMDA, Phencyclidine	Rat	10 µM	-13
Histamine H1	human	10 µM	-3
Nicotinic Acetylcholine	Human	10 µM	-6
Nicotinic Acetylcholine α1, Bungarotoxin	Human	10 µM	21
Opiate µ(OP3, MOP)	Human	10 µM	-2
Potassium Channel [KATP]	Hamster	10 µM	2
Potassium Channel hERG	human	10 µM	1
Prostanoid EP4	human	10 µM	-21
Serotonin (5-Hydroxytryptamine) 5-HT2B	Human	10 µM	-15
Sigma σ1	Human	10 µM	-15
Sodium Channel, Site 2	rat	10 µM	3
